# Applicability of radiomics in interstitial lung disease associated with systemic sclerosis: proof of concept

**DOI:** 10.1007/s00330-020-07293-8

**Published:** 2020-10-06

**Authors:** K. Martini, B. Baessler, M. Bogowicz, C. Blüthgen, M. Mannil, S. Tanadini-Lang, J. Schniering, B. Maurer, T. Frauenfelder

**Affiliations:** 1 Institute of Diagnostic and Interventional Radiology, University Hospital Zurich, University of Zurich, Rämistrase 100, 8091 Zurich, Switzerland; 2 Department of Radiation Oncology, University Hospital Zurich, University of Zurich, Zurich, Switzerland; 3 Center of Experimental Rheumatology, Department of Rheumatology, University Hospital Zurich, University of Zurich, Zurich, Switzerland

**Keywords:** Systemic sclerosis, Pulmonary fibrosis, Artificial intelligence

## Abstract

**Objective:**

To retrospectively evaluate if texture-based radiomics features are able to detect interstitial lung disease (ILD) and to distinguish between the different disease stages in patients with systemic sclerosis (SSc) in comparison with mere visual analysis of high-resolution computed tomography (HRCT).

**Methods:**

Sixty patients (46 females, median age 56 years) with SSc who underwent HRCT of the thorax were retrospectively analyzed. Visual analysis was performed by two radiologists for the presence of ILD features. Gender, age, and pulmonary function (GAP) stage was calculated from clinical data (gender, age, pulmonary function test). Data augmentation was performed and the balanced dataset was split into a training (70%) and a testing dataset (30%). For selecting variables that allow classification of the GAP stage, single and multiple logistic regression models were fitted and compared by using the Akaike information criterion (AIC). Diagnostic accuracy was evaluated from the area under the curve (AUC) from receiver operating characteristic (ROC) analyses, and diagnostic sensitivity and specificity were calculated.

**Results:**

Values for some radiomics features were significantly lower (*p* < 0.05) and those of other radiomics features were significantly higher (*p* = 0.001) in patients with GAP2 compared with those in patients with GAP1. The combination of two specific radiomics features in a multivariable model resulted in the lowest AIC of 10.73 with an AUC of 0.96, 84% sensitivity, and 99% specificity. Visual assessment of fibrosis was inferior in predicting individual GAP stages (AUC 0.86; 83% sensitivity; 74% specificity).

**Conclusion:**

The correlation of radiomics with GAP stage, but not with the visually defined features of ILD-HRCT, implies that radiomics might capture features indicating severity of SSc-ILD on HRCT, which are not recognized by visual analysis.

**Key Points:**

*• Radiomics features can predict GAP stage with a sensitivity of 84% and a specificity of almost 100%.*

*• Extent of fibrosis on HRCT and a combined model of different visual HRCT-ILD features perform worse in predicting GAP stage.*

*• The correlation of radiomics with GAP stage, but not with the visually defined features of ILD-HRCT, implies that radiomics might capture features on HRCT, which are not recognized by visual analysis.*

**Electronic supplementary material:**

The online version of this article (10.1007/s00330-020-07293-8) contains supplementary material, which is available to authorized users.

## Introduction

Interstitial lung disease (ILD) is a well-known complication of systemic sclerosis (SSc) affecting over 60% of patients [1–3] and represents the leading cause of disease-related death [4].

The detection of SSc-ILD is crucial because an early diagnosis of SSc-ILD has important prognostic and therapeutic implications. Novel imaging approaches such as quantitative computed tomography (CT) [5, 6], magnetic resonance imaging (MRI) [7, 8], and nuclear imaging [9, 10] are applied in ILD to provide prognostic, functional, and metabolic information [11]. So far, high-resolution CT (HRCT), a non-invasive, cost-effective, and sensitive technique, remains the gold standard for ILD diagnosis because it is able to detect lung involvement prior to appearance of clinical symptoms and provides prognostic information [12–14]. However, there are many features to determine the presence of ILD and inter-reader variability, especially in unexperienced readers, is an issue.

Most patients with SSc-ILD have mild or stable disease, which does not warrant treatment, only surveillance [15]. However, the high morbidity and mortality of progressive SSc-ILD define the need for early detection for therapeutic intervention. Such a screening modality should combine both high sensitivity and reproducibility.

Radiomics, defined as the conversion of medical images to higher-dimensional data, is a novel research area. Feature extraction is a crucial step in radiomics and comprises the computation of texture, density, and shape from predefined regions of interest (ROIs). Radiomics offers the advantage of an objective quantification of tissue characteristics and enables the detection of abnormalities in radiological images not depicted by routine visual analysis [16–19]. Due to the high objectivity and reliability of data, radiomics shows great potential as support for clinical decision-making [20]. Radiomics has attracted increased attention in recent years, and several studies show that radiomics can be of benefit in terms of prognosis and diagnosis of multiple diseases, especially malignancies [21–23]. In SSc-ILD, to the best of our knowledge, radiomics analyses have not yet been performed.

Currently, no validated single tools are established for staging in SSc-ILD although in clinical practice, a 70% threshold of percentage predicted forced vital capacity (FVC [%]) and extent of fibrosis on HRCT with a threshold of 20% are routinely used [13, 24]. Although most commonly employed, pulmonary function tests as “stand-alone” examination are inferior for diagnostic purposes than HRCT [2]. To overcome the limitations of single factors, several composite scores have been proposed: One of them is the so-called gender, age, and pulmonary function (GAP) score and staging system, developed by Ley et al in 2012 [25]. The system uses four variables: gender (G), age (A), and two pulmonary physiological parameters (P)—FVC [%] and percentage predicted diffusion capacity of the lungs for carbon monoxide (DL_CO_ [%]). The score has been validated in the USA, Italy, and South Korea and showed robust predictive power in patients with chronic ILD [26, 27]. GAP stage is not routinely calculated in SSc-ILD, and visual analysis of ILD criteria on HRCT does not, or not sufficiently, reflect prognosis. We hypothesize that radiomics features might provide important information on disease extent and could potentially influence individual patient management.

In this retrospective pilot study, we aim to evaluate if texture-based radiomics features are able to detect ILD and to distinguish between the different disease stages in patients with SSc-ILD in comparison with mere visual analysis of HRCT.

## Methods

### Patients

Sixty patients (46 females, median age 56 years), who were part of the SSc cohort at the University Hospital Zurich, Switzerland (EUSTAR online Database [pre-BASEC-EK-839, BASEC KEK-Nr.-2016-01515] and VEDOSS online Database [BASEC-Nr.2010-158/5]), fulfilled the ACR/EULAR classification criteria [28], and underwent HRCT (Table 1) between January 2012 and October 2015 with signs of ILD, were retrospectively included in the study. The corresponding image analysis was done retrospectively. Demographic and clinical data, as well as values for pulmonary function tests (PFT), were acquired for each patient (Table 1). The PFT indices included the actual values and the percentage predicted values of a certain age, height, and gender group (%predicted) of forced expiratory volume in 1 s (FEV_1_), forced vital capacity (FVC), total lung capacity (TLC), and diffusion capacity of carbon monoxide (DCLO). In order to make results comparable throughout the study population, %predicted values were used for statistical evaluation. GAP stage was calculated according to Mango et al [25, 29]. Patient characteristics are summarized in Table 1. The retrospective study has been approved by the institutional review board (BASEC-Nr. 2018-02165), and written informed consent was sought from all patients.

**Table 1:** Main patient characteristics. *n* number of patients, *f/m* female/male, *y/n* yes/no, *SD* standard deviation, *mRSS* modified Rodnan skin score, *ILD* interstitial lung disease, *HRCT* high-resolution computed tomography. The PFT indices included the percentage predicted values of a certain age, height, and gender group (%predicted) of forced expiratory volume in 1 s (FEV_1_), forced vital capacity (FVC), total lung capacity (TLC), and diffusion capacity of carbon monoxide (DCLO). Percentage of fibrosis per lung (fibrosis > 20%). *Antibodies comprised anti-centromere antibodies, anti-nuclear antibodies, anti-topoisomerase I antibodies, anti-RNA-polymerase III antibodies, and anti-U1nRNP antibodies. **Immunosuppressive therapy included prednisone, cyclophosphamide, methotrexate, azathioprine, mycophenolate mofetil, d-penicillamine, rituximab, imatinib, and anti-TNF (tumor necrosis factor alpha) inhibitors. ***Expert opinion by echocardiography

Patient information	GAP1	GAP2	*p* value
Demographics			
Number (*n*)	54	6	
Gender (f/m)	48/6	4/2	0.129
Median age (range)	58 (23–82)	68 (54–80)	0.022
Mean mRSS score (range)	5.7 (0–31)	11.7 (0–22)	0.176
Disease subset			
Limited cutaneous	22 (37%)	4 (67%)	0.224
Diffuse cutaneous	10 (17%)	1 (17%)	> 0.999
Sclerodactyly only	17 (28%)	1 (17%)	0.452
Sine scleroderma	4 (7%)	0 (0%)	0.687
Other organ manifestations			
GIT	33 (55%)	4 (67%)	0.791
Renal crisis	0 (0%)	0 (0%)	> 0.999
Pulmonary arterial hypertension***	10 (27%)	3 (50%)	0.076
Cardiac involvement	24 (40%)	1 (17%)	0.190
Auto-abs*	54 (100%)	6 (100%)	> 0.999
Immunomodulatory therapy**	54 (100%)	6 (100%)	> 0.999
Lung function			
FEV_1_% (mean ± SD)	93 ± 16	61 ± 11	< 0.001
FVC% (mean ± SD)	98 ± 18	65 ± 26	0.035
TLC% (mean ± SD)	94 ± 21	57 ± 23	0.013
DCLO% (mean ± SD)	79 ± 19	56 ± 23	0.076
Features of ILD on HRCT			
Pulmonary emphysema (y/n)	5 (8%)	3 (50%)	0.005
Honeycombing (y/n)	5 (8%)	3 (50%)	0.005
Subpleural lines (y/n)	46 (77%)	6 (100%)	0.311
Bronchiectasis (y/n)	19 (32%)	5 (83%)	0.022
Ground-glass opacities (y/n)	25 (42%)	4 (67%)	0.344
Reticular changes (y/n)	46 (77%)	6 (100%)	0.311
Pleural margins (y/n)	31 (52%)	5 (50%)	0.219
Mean coarseness score (± SD)	11.8 (± 3.3)	16.8 (± 3.3)	0.001
Fibrosis > 20% on HRCT (y/n)	8 (13%)	3 (50%)	0.257

### HRCT protocol

All HRCT images were acquired in prone position in full inspiration. HRCT scans were obtained with a 64-slice CT scanner (Somatom Definition AS, Siemens Healthineers). The CT protocol included a topogram and one series in prone position in full inspiration. The following parameters were used for the standard HRCT: tube voltage 120 kV, tube current 30 mAs (reference dose, care dose: on), slice thickness: 1 mm, increment: 0.8 mm, kernel B70. The standard HRCT was reconstructed with iterative reconstruction (SAFIRE) strength 3 [30].

### ILD features on HRCT

**Fig. 1:**
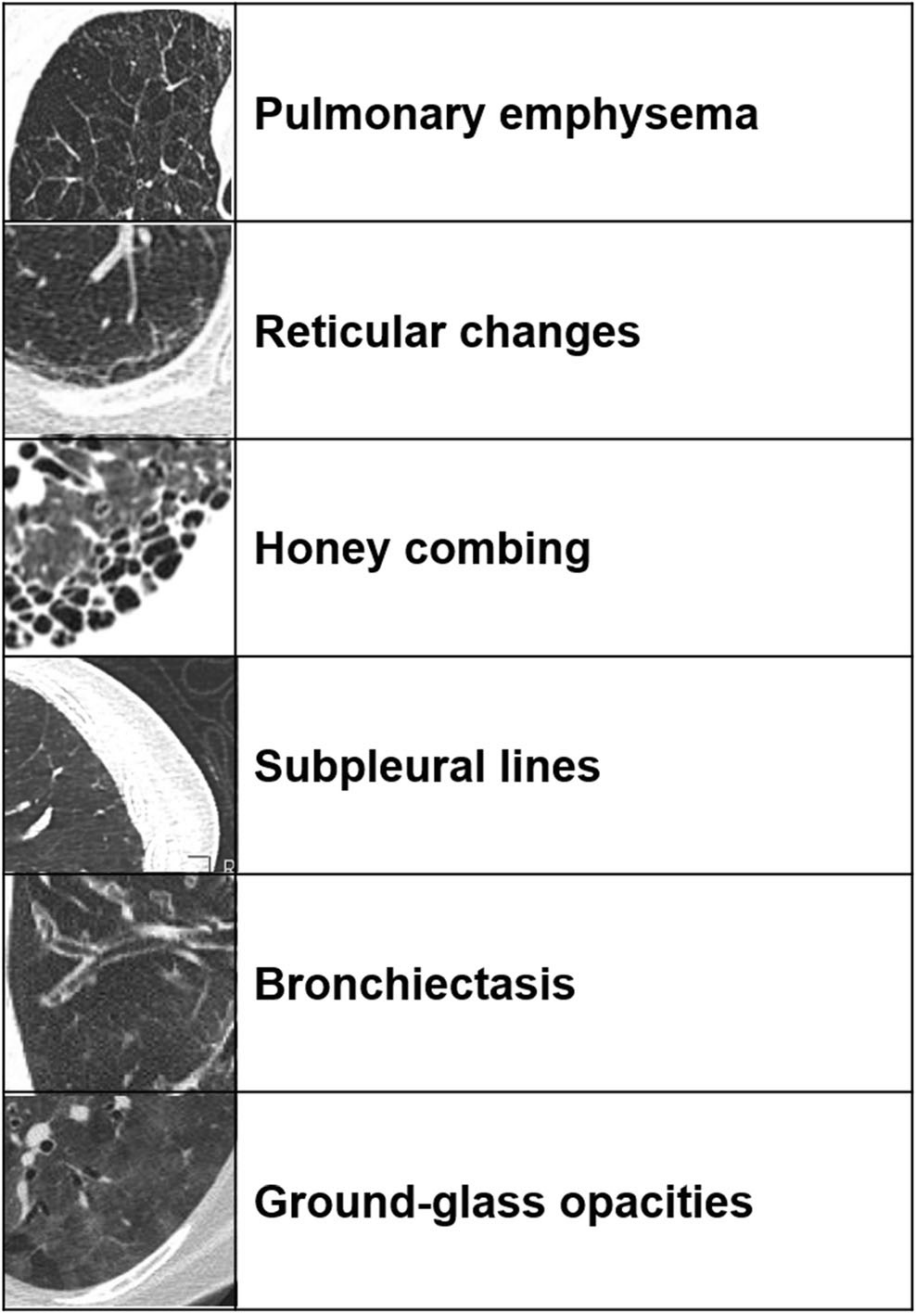
Features of interstitial lung disease (ILD) on high-resolution computed tomography (HRCT)

The readout was performed by two radiologists (T.F. 16 and K.M. 5 years of experience in thoracic imaging) by consensus: If there was disagreement between the two readers, whether an HRCT feature was present or not, re-assessment was performed until consensus was reached. Images where evaluated for the presence of characteristic visual ILD features (yes/no) including pulmonary emphysema, honeycombing, subpleural lines, pleural margins, bronchiectasis, ground-glass opacities, and reticular changes (Fig. 1). A case-by-case evaluation was performed.

Image analysis was performed on a standard picture archiving and communication system workstation (Impax, Version 6.5.5.1033; Agfa-Gevaert) and a high-definition liquid crystal display monitor (BARCO; Medical Imaging Systems).

### Visual assessment of lung fibrosis severity

#### Extent of lung fibrosis

According to Goh et al [13], estimation of disease extent defined as definitely less than 20% (mild disease extent) or definitely more than 20% (severe disease extent) was performed. All sections from the lung apex to the hemidiaphragm were evaluated. In order to keep results specific for visual analysis, we did not include the FVC threshold of 70% proposed by Goh et al [13] in cases with an indeterminate extent of disease on HRCT.

Pulmonary fibrosis was defined as presence of reticular changes, honeycombing, or both.

#### Coarseness of lung fibrosis

The most extensive parenchymal pattern in each lobe was recorded as categorical coarseness grade 0, normal lung; grade 1, ground-glass opacity; grade 2, fine reticulation; grade 3, coarse reticulation; and grade 4, honeycombing. The primary coarseness score represented the sum of coarseness grades (grade 0–4). To remove the effect of pattern extent and prevent the underestimation of coarseness severity in patients, in whom some lobes had no parenchymal abnormality, the score was adjusted proportionally to a six-lobe score [31]:

$$ \mathrm{CS}={\sum}_{1-n}^n\left(\mathrm{CG}\right)/{\mathrm{L}}_{\mathrm{ILD}}\ast 6 $$

where *n* is the number of lobes, CS is the coarseness score, CG is the coarseness grade, and *L*_ILD_ is the number of lobes with ILD.

### Radiomics

#### 3D lung segmentation

We chose to segment only the right lung, since the presence of the heart on the left side potentially makes lung segmentation more difficult and may lead to alteration of results. The right lung of each patient was segmented semi-automatically with dedicated software MIM (Version 6.0, MIM Software Inc.) by setting the Hounsfield unit (HU) values from − 950 to − 150 HU. Where automatically registered borders did not correspond with lung borders, manual corrections were made. The hilar vessels were carefully excluded.

#### Extraction of texture features

Prior to analysis, all images were resampled to isotropic voxels of 2 mm, using linear interpolation [32]. In total, 1116 features were extracted with two bin sizes (10 and 35 HU) corresponding to the following feature classes [33]:

- 4 shape features
- 19 intensity features
- 105 texture features (52 from the gray-level co-occurrence matrix, 5 from the neighborhood gray-tone difference matrix, 32 from the gray-level run length matrix, and 16 from the gray-level size zone matrix)
- 976 wavelet features (coiflet filtering)

Feature descriptions and mathematical definitions were used as described (see the Supplemental Material).

### Data augmentation

Data augmentation was performed using the imbalance package in R (version 3.4.0; R Foundation for Statistical Computing) and applying a majority weighted minority oversampling technique (MWMOTE) (details can be found in the Supplemental Material). After applying the MWMOTE technique, the dataset consisted of an equal number of GAP1 (*n* = 54) and GAP2 (*n* = 54) stage patients. An example of data oversampling and resulting feature values is shown in the Supplemental Material.

### Splitting of the dataset into training and testing datasets

In order to ensure the generalizability of the trained statistical models, the balanced dataset was then randomly split into separate training (*n* = 76 patients, *n* = 38 GAP1 and *n* = 38 GAP2) and testing dataset (*n* = 32 patients, *n* = 16 GAP1 and *n* = 16 GAP2) using a ratio of 0.7:0.3. The entire dimension reduction and feature selection process as further described in the “Results” section was performed only on the training dataset.

### Statistical analysis

Statistical analysis was performed in R (version 3.4.0; R Foundation for Statistical Computing) with RStudio (version 1.0.136; RStudio). R packages used for statistical analysis are described in the Supplemental Material. All continuous data are given as means ± standard deviation. Categorical variables are expressed as frequencies or percentages. A two-tailed *p* value of < 0.05 was considered to indicate statistical significance. Testing for group differences was performed by using Wilcoxon’s signed-rank tests and Friedman’s test after assessing normal distribution of the data. The chi-squared test was used to compare categorical parameters.

For selecting variables that allow classification of GAP stages 1 and 2, single and multiple logistic regression models were fitted and compared by using the Akaike information criterion (AIC). The misclassification rate of these models was assessed by using 10-fold cross-validation. The diagnostic accuracy of optimal predictive parameters was evaluated from the area under the curve (AUC) from receiver operating characteristic (ROC) analyses, and diagnostic sensitivity and specificity were calculated.

Similarly, predictive value of ILD-HRCT features for the GAP stage was tested.

## Results

### Visual assessment of HRCT

In 17 out of the 60 cases, readers disagreed about the presence of ILD features. In these cases, disagreement was resolved in consensus reading.

Eight patients showed pulmonary emphysema (13%), eight honeycombing (13%), 52 subpleural lines (87%), 24 bronchiectasis (40%), 29 ground-glass opacities (48%), 52 reticular changes (87%), 36 pleural margins (60%), and 11 fibrosis involving more than 20% of the lung parenchyma (18%). Mean coarseness score was 12.3 (SD ± 3.6).

**Fig. 2:**
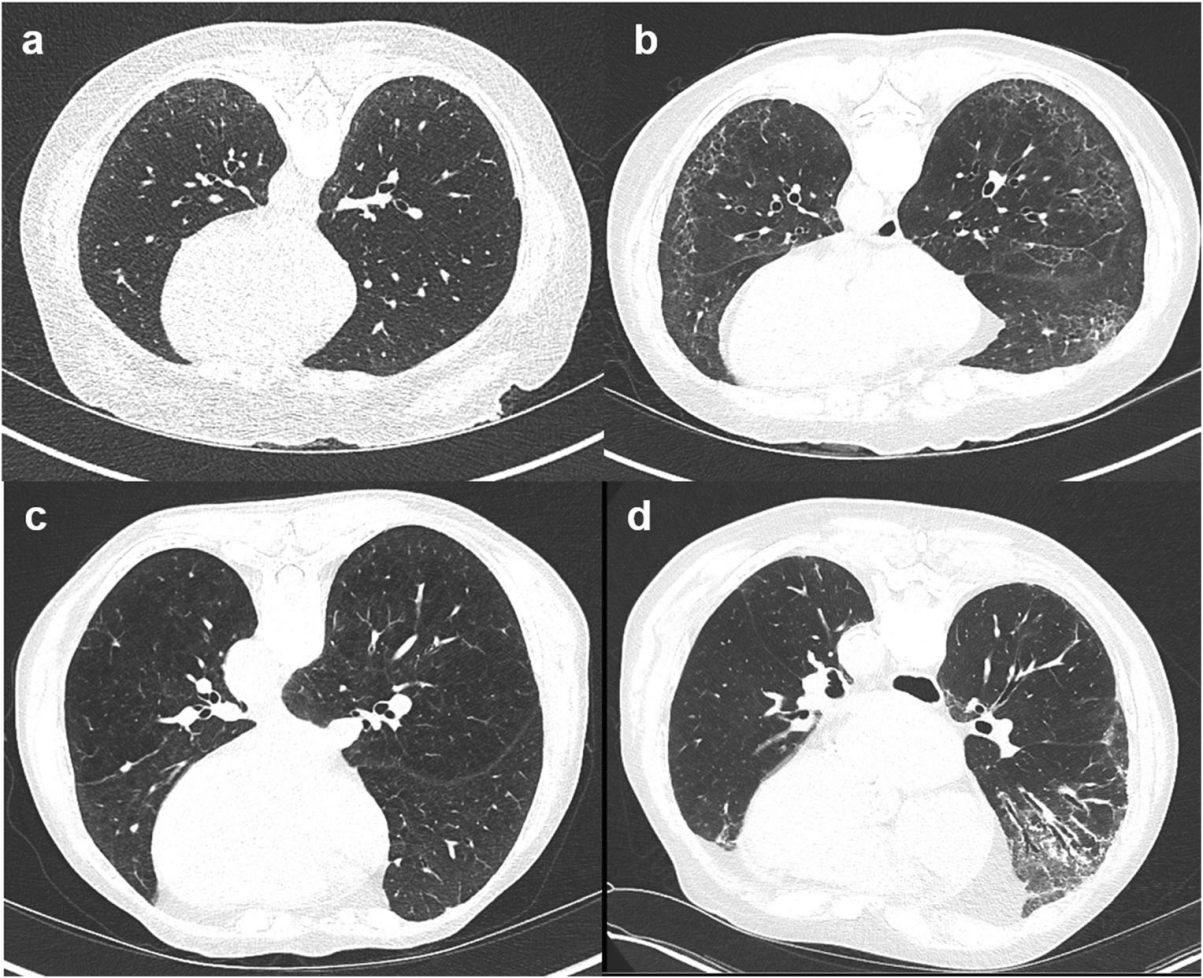
Varying grades of fibrosis within the same GAP stages. Patients with **a**, **b** GAP stage 1 and **c**, **d** GAP stage 2. **a** A 43-year-old female patient with GAP1 shows subpleural reticulations, subpleural lines, and some ground-glass opacification—overall less than 10% of fibrosis. **b** A 56-year-old female patient with GAP1 shows basal and subpleural reticulations, ground-glass opacities, and subpleural lines accounting for more than 20% of lung fibrosis. **c** A 69-year-old female patient with GAP2 shows only discrete subpleural lines. No other signs of fibrosis are visualized. **d** An 80-year-old male patient with GAP2 showing subpleural reticulations accompanied by ground-glass opacification, subpleural lines, and pleural margins in the right lung

For detailed information and distribution of the features among GAP stages, please refer to Table 1 and Fig. 2.

Highest AUC could be obtained when combining honeycombing, emphysema, and bronchiectasis in a model, which resulted in an AUC of 0.86 with a sensitivity of 100% and a specificity of 63%. When performing ROC analysis, the AUC for predicting GAP stage with extent of fibrosis (fibrosis > 20%) is 0.606 (95% confidence interval 0.543–0.791, *p* = 0.145) with a sensitivity of 50% and a specificity of 85.2%.

When performing ROC analysis for coarseness score of fibrosis, the AUC for predicting GAP stage reached 0.863 (95% confidence interval 0.703–1.000, *p* = 0.004) with a sensitivity of 83% and a specificity of 74%. Differences between predicting ROC curves with extent of fibrosis versus coarseness of fibrosis were not statistically significant (*p* = 0.057).

### Radiomics

#### Dimension reduction and radiomics feature selection for classification of GAP1 versus GAP2 stage

**Fig. 3:**
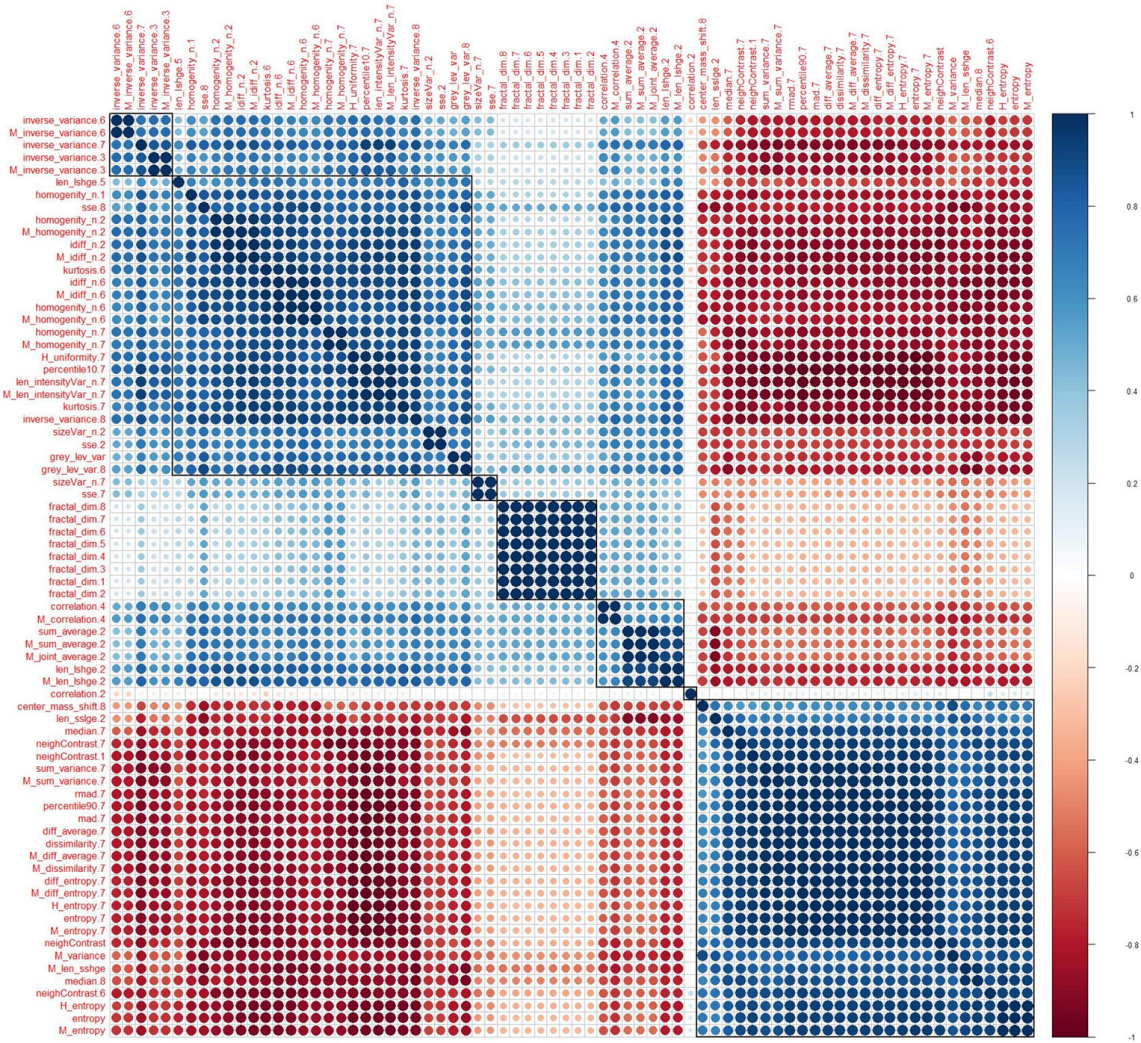
Correlogram illustrating auto- and cross-correlations of the 73 most important features in differentiating GAP1 and GAP2 stages. Features were reordered after hierarchical clustering for visualizing different feature clusters. Six clusters of radiomics features became apparent (rectangular boxes; the first two clusters were visually assumed to belong to the same cluster). Blue circles indicate positive correlation, and red circles negative correlation. The larger the circle and the darker the color, the higher is the correlation between two variables

Radiomics feature selection and dimension reduction were performed on the augmented training dataset. After normalization of all numeric features using z-score standardization, features were fed into the Boruta dimension reduction and feature elimination algorithm as described previously [25, 26], resulting in the selection of 73 features, which were considered most important for classification accuracy. Since the Boruta algorithm does not account for collinearity in the data, a correlation matrix was calculated in a next step in order to detect clusters of highly correlated features (defined as Pearson’s *r* ≥ .60; Fig. 3). After visualization of each single parameter in box and whisker plots and random forest models fitted separately on each of the six detected correlation clusters, only one feature from each cluster with the highest Gini index and visually the best separation between the two groups (“GAP1” and “GAP2” stage) was selected for further analysis. At the end of the multistep dimension reduction process, the six most important and independent features were selected for further statistical analyses: M_homogenity_n.LHL, neighContrast.LHL, fractal_dim.LLL, M_correlation.HLL, M_correlation.HHL, and sizeVar_n.LLH.

#### Training of statistical models for classification of GAP1 versus GAP2 stage

**Fig. 4:**
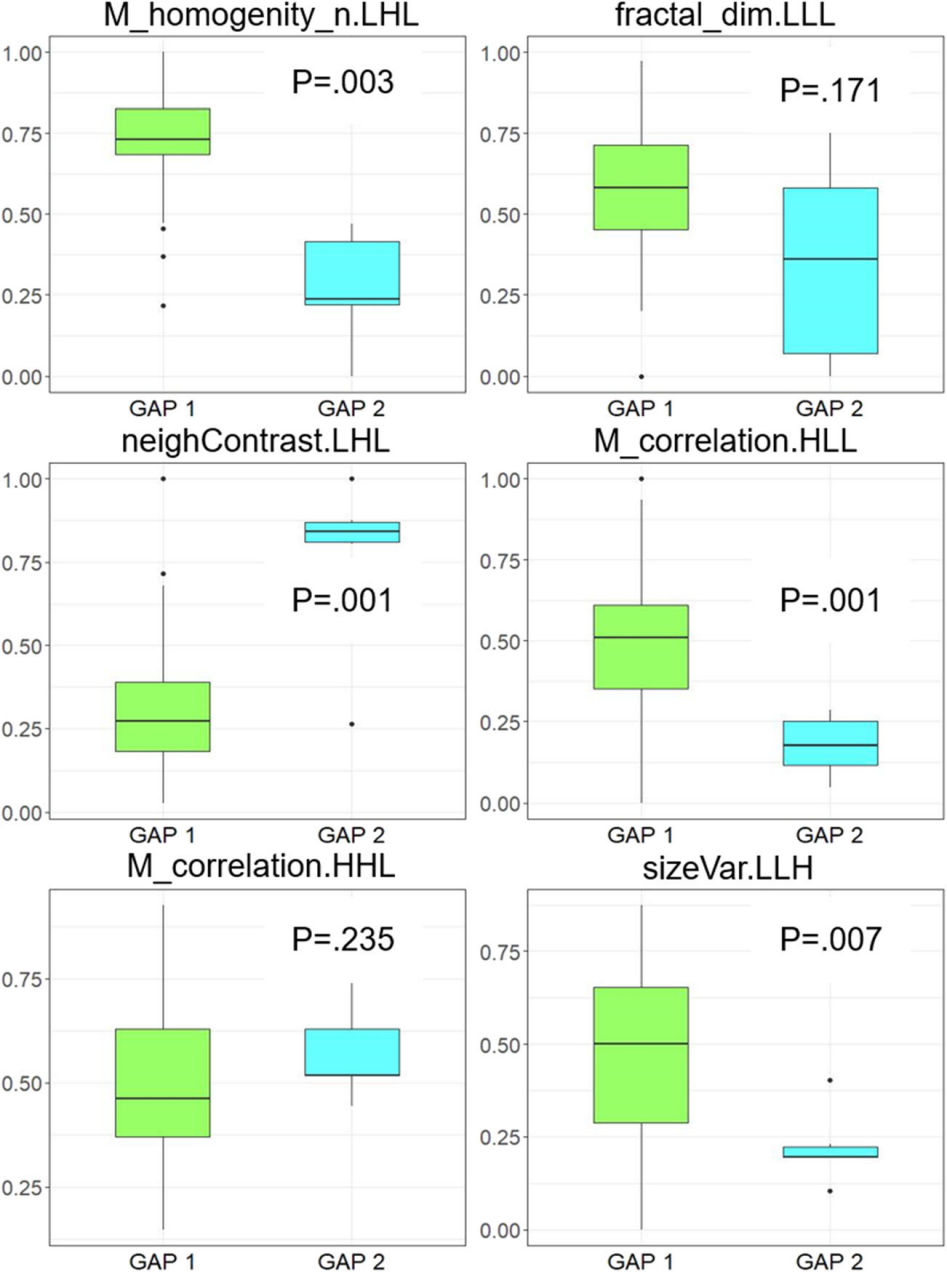
Box and whisker plots show differences of the 6 most important radiomics features selected during the multistep dimension reduction and feature selection process between GAP1 and GAP2 patients. Center line in each box represents median. Lower and upper limits of each box represent the 25th and 75th percentiles, respectively. Whiskers extend to most extreme observations within 25th and 75th percentiles ± 1.5 × interquartile range. Observations outside these whiskers are shown as dots

In the original non-augmented dataset, values for M_homogenity_n.LHL, M_correlation.HLL, and sizeVar_n.LLH were significantly lower in patients with a GAP stage of 2 when compared with those in patients with a GAP stage of 1 (*p* = 0.003, 0.001, and 0.007, respectively; Fig. 4 and Table 2). In contrast, values for neighContrast.LHL were significantly higher in patients with a GAP stage of 2 (*p* = 0.001). No significant differences were observed for fractal_dim.LLL and correlation.HHL, although the difference for fractal_dim.LLL reached statistical significance in the augmented dataset.

**Table 2:** Results of radiomics. *GAP stage* gender, age, and pulmonary function stage, *n* number of patients

Variable	GAP1 (*n* = 54)	GAP2 (original; *n* = 6)	GAP2 (augmented; *n* = 54)	*p* value GAP1 versus GAP2 (original)	*p* value GAP1 versus GAP2 (augmented)
M_homogenity_n.LHL	0.73 ± 0.14	0.33 ± 0.27	0.25 ± 0.18	.003	< 0.001
neighContrast.LHL	0.31 ± 0.17	0.77 ± 0.26	0.81 ± 0.15	.001	< 0.001
fractal_dim.LLL	0.58 ± 0.20	0.39 ± 0.39	0.36 ± 0.25	.171	< 0.001
M_correlation.HLL	0.49 ± 0.21	0.18 ± 0.09	0.16 ± 0.07	.001	< 0.001
correlation.HHL	0.49 ± 0.20	0.57 ± 0.11	0.54 ± 0.07	.235	0.057
sizeVar_n.LLH	0.47 ± 0.22	0.22 ± 0.10	0.21 ± 0.06	.007	< 0.001

Single and multiple logistic regression models were fitted on the training dataset and compared according to their AIC. In single logistic regression models, M_homogenity_n.LHL and neighContrast.LHL showed the lowest AIC with 21.13 and 23.81, respectively (fractal_dim.LLL: 98.37, M_correlation.HLL: 41.50, correlation.HHL: 107.16, and sizeVar_n.LLH: 75.59). Results of the corresponding ROC analyses for the training, testing, and the original (non-augmented) datasets are shown in Table 3.

**Table 3:** Diagnostic performance of radiomics features and visual assessment of HRCT features. *GAP stage* gender, age, and pulmonary function stage, *n* number of patients, *AUC* area under the curve with bootstrapped 95% confidence intervals (CI)

**Variable**	**GAP1 versus GAP2 (augmented training set)**			**GAP1 versus GAP2 (augmented testing set)**			**GAP1 versus GAP2 (original dataset)**		
	**AUC [95% CI]**	**Sensitivity (%)**	**Specificity (%)**	**AUC [95% CI]**	**Sensitivity (%)**	**Specificity (%)**	**AUC [95% CI]**	**Sensitivity (%)**	**Specificity (%)**
M_homogenity_n.LHL	0.99 [0.98–1.00]	97	95	0.91 [0.80–1.00]	100	75	0.87 [0.65–1.00]	83	94
neighContrast.LHL	0.99 [0.98–1.00]	97	95	0.88 [0.75–1.00]	88	88	0.90 [0.73–1.00]	83	98
fractal_dim.LLL	0.72 [0.60–0.84]	50	95	0.87 [0.73–1.00]	75	94	0.67 [0.35–0.99]	50	94
M_correlation.HLL	0.95 [0.89–1.00]	100	90	0.88 [0.71–1.00]	100	81	0.91 [0.83–0.99]	100	82
correlation.HHL	0.60 [0.45–0.75]	97	53	0.63 [0.43–0.84]	100	38	0.65 [0.49–0.81]	100	44
sizeVar_n.LLH	0.85 [0.75–0.95]	87	82	0.86 [0.70–1.00]	100	81	0.84 [0.71–0.96]	83	82
M_homogenity_n.LHL + neighContrast.LHL	0.99 [0.98–1.00]	95	95	0.90 [0.79–1.00]	100	69	0.89 [0.71–1.00]	83	98
neighContrast.LHL + M_correlation.HLL	1.00 [0.99–1.00]	100	97	0.92 [0.79–1.00]	100	88	0.96 [0.90–1.00]	84	99
**Variable**		**GAP1 versus GAP2 (original dataset)**							
		**AUC [95% CI]**			**Sensitivity (%)**		**Specificity (%)**		
Ground.Glass		0.60 [0.39–0.82]			67		54		
Pleural.margins		0.63 [0.45–0.81]			83		43		
Subpleural.lines		0.57 [0.53–0.62]			100		15		
Honey.combing		0.70 [0.48–0.93]			50		91		
Emphysema		0.70 [0.48–0.93]			50		91		
Bronchiectasis		0.74 [0.57–0.92]			83		65		
Ground.Glass + Pleural.margins + Subpleural.lines + Honey.combing + Emphysema + Bronchiectasis		0.86 [0.74–0.98]			100		65		
Honey.combing + Emphysema + Bronchiectasis		0.86 [0.75–0.98]			100		63		

**Fig. 5:**
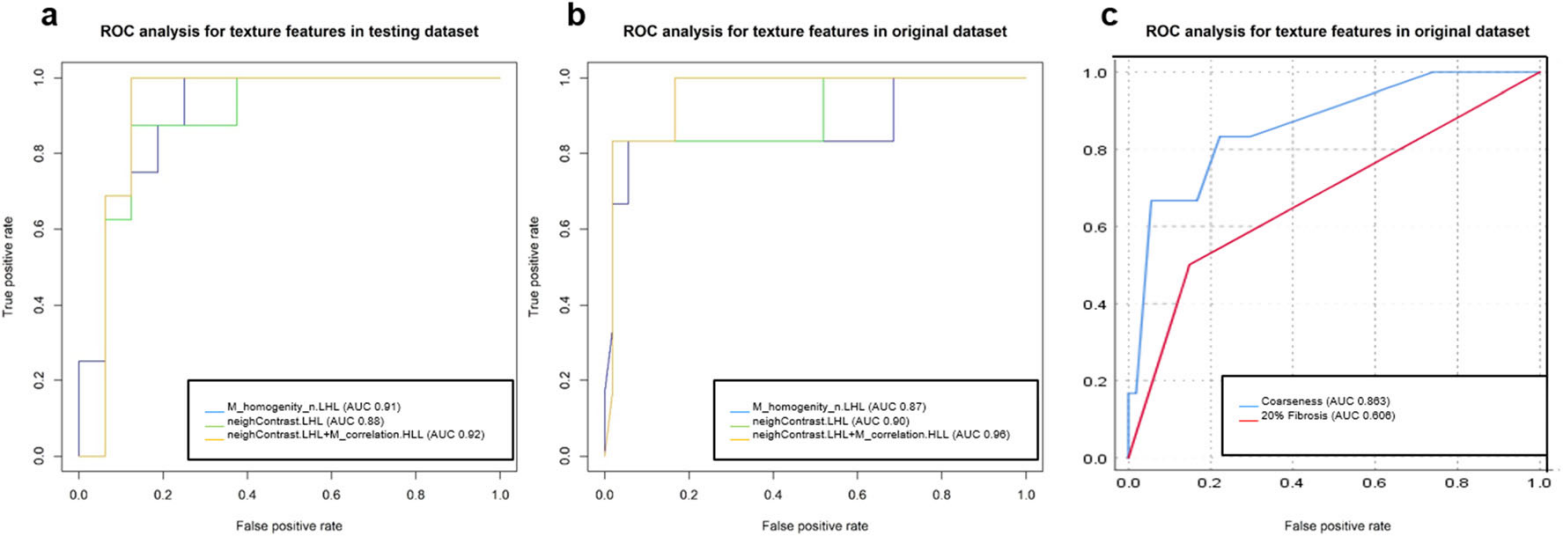
Graphs show receiver operating characteristics (ROC) analyses for the best classifying models of GAP1 versus GAP2 stage. ROC analysis indicates accuracy, sensitivity, and specificity of the best performing models applied on the (**a**) independent testing dataset and on the (**b**) original (non-augmented) dataset. Panel **c** showing ROC analyses for classifying GAP stage with visual analysis of extent of fibrosis (fibrosis > 20%) and coarseness of fibrosis

Combining M_homogenity_n.LHL and neighContrast.LHL in a model resulted in a higher AIC (21.94) and showed collinearity of the two features without significant improvement of diagnostic sensitivity and specificity. The combination of neighContrast.LHL and M_correlation.HLL in a multivariable model finally resulted in the lowest AIC of 10.73 with an AUC of 1.00, 100% sensitivity, and 97% specificity in the training dataset; an AUC of 0.92, 100% sensitivity, and 88% specificity in the test dataset; and an AUC of 0.96, 84% sensitivity, and 99% specificity in the original dataset (Fig. 5 and Table 3).

Ten-fold cross-validation of this model in the independent test dataset resulted in a cross-validation estimate of an accuracy of 0.88 (95% confidence interval 0.71–0.97).

## Discussion

HRCT imaging together with PFT is currently the gold standard for a cost-effective, non-invasive assessment of ILD [34]. However, features to determine the presence of ILD are manifold and inter-reader variability, especially in unexperienced readers, is an issue. Radiomics, in contrast, is an objective imaging-based tool that enables a more detailed and reliable quantitative assessment of lesion characteristics, which is not hampered by subjective image interpretation and experience of the reader as in visual analysis.

In this study, we were able to show that radiomics features can predict GAP stage with a sensitivity of 84% and a specificity of almost 100%. Extent of fibrosis on HRCT and a combined model of different visual HRCT-ILD features performed worse in predicting GAP stage. We believe that this is due to the high inter-reader variability, even in expert radiologists, in determining the presence and severity of ILD features.

Since the dataset in our patient cohort was imbalanced regarding the distribution of the two classes with 54 patients in GAP1 stage, but only six patients in GAP2 stage (imbalanced ratio: 0.11)—thereby reflecting the prevalence of GAP1 versus GAP2 stages in our cohort of SSc patients—we performed a data augmentation step in order to achieve better class balance and to avoid model overfitting before further evaluation. This data augmentation technique does not affect the reliability of the statistical evaluation, and results have been additionally tested on the original dataset.

Extracted radiomics features can be divided into four groups, namely (1) first-order histogram-based features, (2) co-occurrence matrix-based features, (3) multiscale features, and (4) other features [35, 36]. The latter are part of a specific group of features that are related to neighborhood gray-tone difference matrix (GTDM) [35, 37, 38]. The GTDM is based on measuring the difference between the intensity level between each voxel and its neighboring voxels, resulting in features to resemble the human perception of the image. Homogeneity (M_homogenity_n.LHL) reflects the homogeneity of image textures and scales the local changes of image texture. High values of homogeneity denote the absence of intra-regional changes and locally homogenous distribution in image textures [39]. Fractal features (fractal_dim.LLL) provide important spatial information. Contrast (neighContrast.LHL) and correlation (M_correlation.HLL and correlation.HHL) rely on perceptual attributes of texture in terms of spatial changes in intensity or dynamic range of intensity [35, 37, 38]. In our study, the combination of neighContrast.LHL and M_correlation.HLL in a multivariable model resulted in an AUC of 0.92, 100% sensitivity, and 88% specificity in the test dataset and an AUC of 0.96, 84% sensitivity, and 99% specificity in the original dataset. AUC of the ROC curve for percentage of fibrosis was significantly worse in predicting GAP stage, and also, a model combining different HRCT-ILD features performed less well than radiomics did. These findings raise the question, if radiomics is able to capture features on HRCT which are not perceptible by the radiologist with the naked eye?

Radiomics has attracted increased attention in recent years, and several studies show that radiomics can be of benefit in terms of prognosis and diagnosis of multiple diseases, especially malignancies [21–23]. These studies have shown that radiomics features show great potential to serve as surrogate imaging markers for tissue biopsies [40] and reliably predict outcome [41–44] and drug response [45, 46]. Currently, there are different approaches for the evaluation of HRCT, namely (1) visual analysis, (2) semiquantitative analysis, and (3) quantitative analysis or automated approaches using artificial intelligence. While sheer visual analysis suffers from a relatively high inter-observer variability [47, 48], semiquantitative and quantitative analyses (such as densitometric analysis) have the potential to overcome the drawbacks of subjective visual assessment of CT images and have also been shown to correlate with therapeutic response outperforming qualitative analysis [48].

In the past decade, radiomics gained importance in medical imaging. Unlike computer-aided detection (CAD) systems, which are directed toward delivering a single answer (i.e., presence of a lesion or cancer), radiomics is a process designed to extract a large number of quantitative features from digital images, which are subsequently mined for hypothesis generation and testing. Recent data from non-malignant lung diseases suggest that the texture-based analysis of CT data might outperform the currently used visual and/or histogram measures for diagnosis and staging [49–51]. The process of radiomics-based stratification of data provides a far more detailed characterization of phenotypes than current criteria can.

Compared with other studies [52], we did not train the algorithm to recognize specific patterns or features, such as honeycombing or bronchiectasis. We trained the system to find an algorithm to differentiate between the different GAP stages. With this approach, we omitted to use pattern-based classifications coming from known guidelines for pulmonary fibrosis, as this might not reflect the activity of the disease and might narrow the diagnosis. By just providing lung function, age and gender as input parameters, the validation of the algorithm is quite open and thus, best-discriminating radiomics features might come from feature groups that are not per se visible or quantifiable by the radiologist.

At present, data on radiomics in ILD are limited. The accumulating results, however, are promising and underline the great potential of radiomics in HRCT for detection and staging. In the future, the use of radiomics in SSc-ILD management could be expanded to support treatment decisions. Future studies integrating both radiomics and tissue-based molecular information, however, will be needed to assess whether radiomics reflect the underlying pathophysiology and thereby allow distinguishing inflammatory from fibrotic processes. This would be the prerequisite for treatment guidance toward anti-inflammatory or anti-fibrotic drugs in the individual patient.

The limitations of this study include as follows: firstly, the GAP staging system consists of three stages (low, intermediate, high). We only have patients with GAP stages 1 and 2 in our cohort and the percentage of patients with GAP stage 2 is relatively small, thereby reflecting the prevalence of GAP1 versus GAP2 stage in our clinical population. We performed a data augmentation step in order to achieve better class balance and to avoid model overfitting. Secondly, we only evaluated data from one institution acquired with one CT scanner. Since differences in scanning parameters such as type of CT scanner, tube voltage, tube current, reconstruction kernel, and contrast agent may influence the results of quantitative analysis, our approach might need to be adapted for future use with other scanners and protocols. Further studies with higher patient numbers, on other scanners, are needed to validate our findings and to investigate potential outcome predictors in a longitudinal study setting. Thirdly, we chose the right lung for image evaluation. Even though evaluation of the left site in our patient population (see Supplemental material) showed comparable results between the two sides, we prefer to use the right lung for image evaluation, since the left lung, due to the proximity of the left lower lobe and lingula to the heart, might be more prone to motion artifacts due to cardiac pulsation and might therefore deliver less robust results. We acknowledge that in cases with asymmetrical lung involvement, this approach might alter the results. Finally, lung segmentation was performed semiautomatically. This approach gave us the opportunity to correct datasets, where automatically registered borders did not correspond with lung borders.

## Conclusion

The correlation of radiomics with GAP stage, yet not with the visually defined features of ILD-HRCT, implies that radiomics might capture features indicating severity of SSc-ILD on HRCT, which are not recognized by visual analysis.

The texture-based radiomics features identified in this pilot study will pave the way for the assessment whether texture-based radiomics signatures may be valuable tools for computer-aided decision-making in imaging.

## Electronic supplementary material

ESM 1 (DOCX 194 kb)

## Abbreviations

GAP stage: Gender, age, and pulmonary function stage
HRCT: High-resolution computed tomography
ILD: Interstitial lung disease
SSc: Systemic sclerosis
