# Supplementary material for: Applicability of radiomics in interstitial lung disease associated with systemic sclerosis: proof of concept
Source: Eur Radiol. 2020 Oct 6;31(4):1987–98. doi: 10.1007/s00330-020-07293-8 (PMC7979612; doi:10.1007/s00330-020-07293-8)
Supplement: Supplementary file 1 — (DOCX 194 kb) [file 330_2020_7293_MOESM1_ESM.docx]

# **R package**

Statistical analysis was performed in R (1) (version 3.4.0; R Foundation for Statistical Computing) with RStudio (2) version 1.0.136; RStudio).

### Further Statistical Analyses

Statistical analysis was performed in the open-source statistics package R (3) (version 3.4.0; R Foundation for Statistical Computing, Vienna, Austria) with RStudio (4) (version 1.0.136; RStudio, Boston, Mass) (13), using the packages tidyverse (5) for data cleansing and reformatting, ggplot2 (6) and gridExtra (7) for graphical visualization, caret (8) for feature normalization, imbalance (9) for data oversampling, Boruta (10) for texture feature selection, corrplot (11) for visualization of correlation matrices, randomForest (12) for fitting single classification trees, Epi (13) and ROCR (14) for receiver operating curve (ROC) analysis, and pastecs (15) for descriptive statistics.

## **References**

1. R Core Team R: A Language and Environment for Statistical Computing, Vienna, Austria.
2. RStudio Team (2015) RStudio: Integrated Development Environment for R. RStudio, Inc., Boston, MA.
3. R Core Team. R: A language and environment for statistical computing. Vienna, Austria: R Foundation for Statistical Computing, 2017.
4. RStudio Team. RStudio: Integrated Development for R. 1.0.136 ed. Boston, MA, USA2016.
5. Hadley Wickham (2017). tidyverse: Easily Install and Load the 'Tidyverse'. R package version 1.2.1. <https://CRAN.R-project.org/package=tidyverse>
6. Wickham H. ggplot2: Elegant Graphics for Data Analysis. Springer-Verlag New York, USA, 2009.
7. Baptiste Auguie (2017). gridExtra: Miscellaneous Functions for "Grid" Graphics. R package version 2.3. <https://CRAN.R-project.org/package=gridExtra>
8. Max Kuhn. Contributions from Jed Wing, Steve Weston, Andre Williams, Chris Keefer, Allan Engelhardt, Tony Cooper, Zachary Mayer, Brenton Kenkel, the R Core Team, Michael Benesty, Reynald Lescarbeau, Andrew Ziem, Luca Scrucca, Yuan Tang, Can Candan and Tyler Hunt. (2019). caret: Classification and Regression Training. R package version 6.0-84. <https://CRAN.R-project.org/package=caret>
9. Ignacio Cordón, Salvador García, Alberto Fernández and Francisco Herrera (2018). imbalance: Preprocessing Algorithms for Imbalanced Datasets. R package version 1.0.0. <https://CRAN.R-project.org/package=imbalance>
10. Kursa MB, Rudnicki WR. Feature Selection with the {Boruta} Package. Journal of Statistical Software. 2010;36(11):1-13.
11. Wei T, Simko V. corrplot: Visualization of a Correlation Matrix. R package version 0.77 ed2016.
12. A. Liaw and M. Wiener (2002). Classification and Regression by randomForest. R News 2(3), 18--22.
13. Bendix Carstensen, Martyn Plummer, Esa Laara, Michael Hills (2019). Epi: A Package for Statistical Analysis in Epidemiology. R package version 2.38. URL <https://CRAN.R-project.org/package=Epi>
14. Sing T, Sander O, Beerenwinkel N, Lengauer T (2005). “ROCR: visualizing classifier performance in R.” _Bioinformatics_, *21*(20), 7881. <URL:<http://rocr.bioinf.mpi-sb.mpg.de>>.
15. Philippe Grosjean and Frederic Ibanez (2018). pastecs: Package for Analysis of Space-Time Ecological Series. R package version 1.3.21. https://CRAN.R-project.org/package=pastecs

# **Data augmentation**

Data augmentation was performed using the imbalance package (1) in R (2) (version 3.4.0; R Foundation for Statistical Computing) and applying a Majority Weighted Minority Oversampling Technique (MWMOTE). SMOTE is a classic algorithm which generates new examples by filling empty areas among the positive instances. It updates the training set iteratively. However, it has a main limitation: it does not detect noisy instances. MWMOTE tries to overcome this problem by intending to give higher weight to borderline instances and undersizing minority cluster instances and examples near the borderline of the two classes.

After applying the MWMOTE technique, the dataset consisted of an equal number of GAP1 (n = 54) and GAP2 (n = 54) stage patients. An example of data oversampling and resulting feature values is shown in Figure 1.


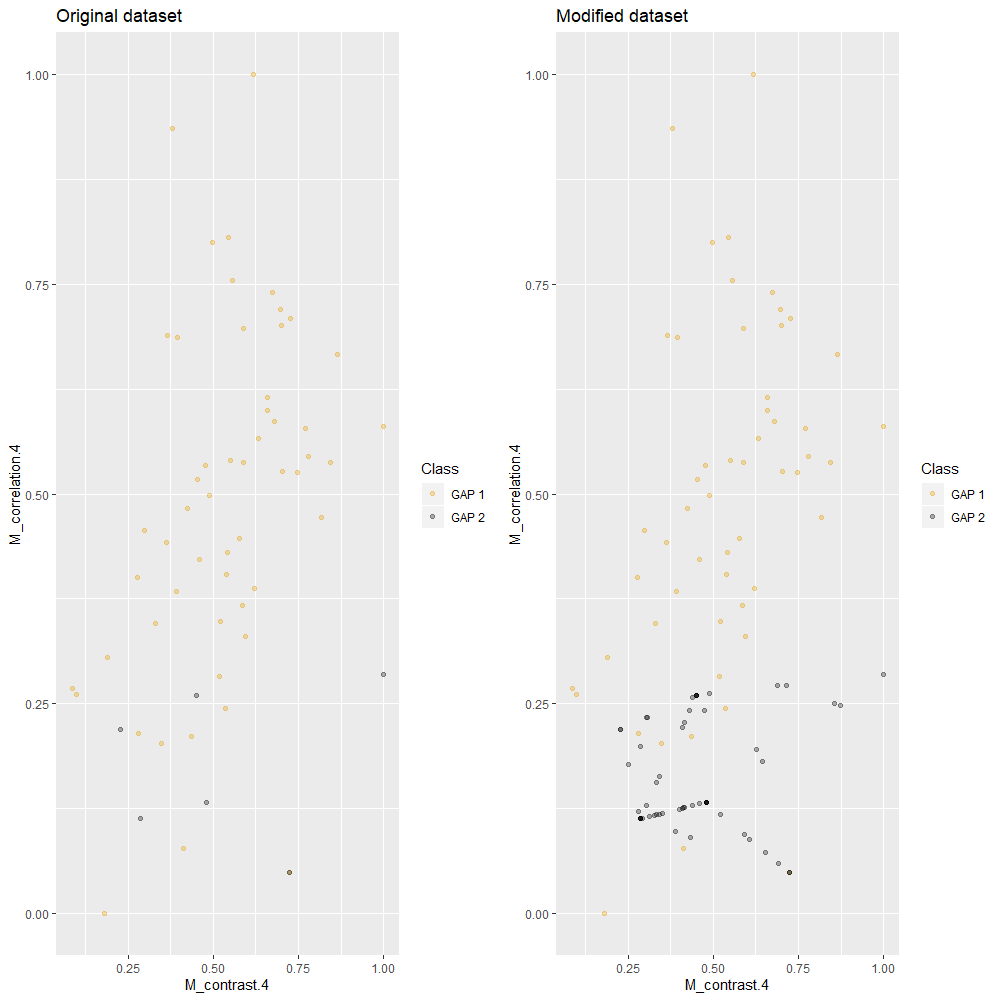
**Figure 1:** Example of data oversampling. Value distribution of the feature M_correlation.4 for GAP stage 1 (yellow) and GAP stage 2 (grey) in the original dataset and the modified (augmented) dataset.

## **References**

1. Ignacio Cordón, Salvador García, Alberto Fernández and Francisco Herrera (2018). imbalance: Preprocessing Algorithms for Imbalanced Datasets. R package version 1.0.0. <https://CRAN.R-project.org/package=imbalance>.
2. R Core Team R: A Language and Environment for Statistical Computing, Vienna, Austria.

# **Z-Rad**

Z-Rad is an in-house developed radiomics software implemented at the Department of Radiation Oncology, University Hospital Zurich.

## **Software**

The software consists of two main modules: a post-processing module to prepare the image data for radiomics calculation and a radiomics calculation module for the effective calculation of radiomic features.

### **Input data**

Z-Rad offers radiomic feature extraction from different medical image modalities, such as: computed tomography, positron emission tomography, magnetic resonance tomography and perfusion computed tomography. It is fully DICOM compatible, handling the medical imaging scans as well as structure set with predefined region of interest (ROI).

### **Post-processing module**

Prior to the calculation of radiomic features from the ROI, Z-Rad allows to resample images and ROI to a predefined spatial resolution using trilinear interpolation *(IBSI p. 5)* [1]*.* The resampled images and structure sets are saved in DICOM format.

### **ROI mask extraction**

The contour points are translated to the voxel coordinates of the analyzed image. Next, those voxels are used to find a contour polygon and points inside this polygon using the pointPolygonTest function from Python cv2 library (version 2.4.6). Next, the image-specific coordinates of voxels inside this polygon are extracted.

### **Image discretization**

Two image discretization methods are implemented according to the recommendation of IBSI (*p. 12)*:

1. Fixed bin size: e.g. 10 HU
2. Fixed number of bins: the grey level value range is divided into e.g. 64 bins

### **Radiomics calculation module**

For the radiomic feature calculation, please refer to the feature definition section. For CT imaging, Hounsfield units (HU) can be manually restricted to a particular HU range, i.e. to exclude bone or air structures from the region of interest. For the PET module, conversion to the standardized uptake value is optional. For the MR module, intensities can be normalized using a linear relationship between two normal tissue ROIs (e.g. muscle and fat).

## **Code**

Z-Rad is implemented in Python programming language 3.7. It utilizes following python packages:

- Numpy >= v1.14.0
- Scipy >= v1.0.0
- Matplotlib >= v2.0.0
- Pydicom >= v1.3.0
- WxPython >= v4.0.6
- VTK >= v8.1.2
- OpenCV >= v4.1.2
- PyWavelets >= v1.1.1

## **Standard features definition**

Z-Rad calculates radiomic features, which can be categorized into four main types: shape, histogram based, texture based and filtered-based (wavelet) features. Features were defined according to the image biomarker standardisation initiative (IBSI, version 9)[1]. The deviations from the IBSI recommendation and additional features are described below. Additionally, detailed information can be found on https://medical-physics-usz.github.io/

### **Shape**

- $median thickness$ - median of distances of each voxel in the region of interest to its surface, calculated using distance transform
- $SD thickness$ - standard deviation of distances of each voxel in the region of interest to its surface
- $maximum 3D diameter$ - the largest pairwise Euclidian distance between voxels of the region of interest
- $fractal dimension$ - calculated using box contouring technique and fixed grid scans excluding the voxels with ‘not a number’ value [2].

$$fractal dimension= -\frac{\ln\left( N\left( r \right) \right)}{\ln\left( r \right)}-I$$

where: $r$ - size of the contouring box, $N(r)$ - number of boxes of size r containing at least one voxel, which belongs the studied structure, $I$ - intercept.

### **Texture matrices**

**The Gray Level Co-occurrence Matrix** *(IBSI p. 57)*

The parameters from the Gray Level Co-occurrence Matrix [3] are calculated in all 26 directions with a distance of one voxel. The final parameters are the average of all directions or are calculated on matrix merged over all directions. If one of the voxels has a ‘not a number’ value the pair was not taken into account in the calculations. Let $P_{ij}$ denotes the $\left( i,j \right)$ entry in the Gray Level Co-occurrence Matrix, $N_{g}$ - number of gray tones in a studied structure, $P_{xi}=\sum_{j=1}^{N_{g}} P_{ij}$, $P_{yj}=\sum_{i=1}^{N_{g}} P_{ij}, P_{x+y}\left( k \right)=\sum_{j=1}^{N_{g}} \sum_{i=1}^{N_{g}} P_{ij},$ where $k=i+j$, $P_{x-y}\left( k \right)=\sum_{j=1}^{N_{g}} \sum_{i=1}^{N_{g}} P_{ij},$ where $k= \left| i-j \right|$.

- $maximal correlation coefficient= \sqrt{second largest eigenvalue of \sum_{k=1}^{N_{g}} \frac{P_{ik}P_{jk}}{P_{xi}P_{ji}}}$

**The Gray Level Distance Zone Matrix** *(IBSI p. 80)*

Missing internal voxels: in the IBSI implementation ‘…the distance map of the GLDZM is affected by the definition of what constitutes the ROI surface. The particular surface used here should match the one used for calculating morphological features. This includes the same treatment of internal missing volumes.’ However, in the Z-Rad implementation the same mask is used as for the calculation of texture features, including e.g. exclusion of voxels with high intensities removed with HU thresholding.

### **Wavelet**

The filter-based features are not standardized within the IBSI (as of November 2019). In the Z-Rad, the wavelet transform is performed in 3D using Coiflet (‘coif1’) function from python package PyWavelets. The decimated wavelet transform (with a downsample factor of 2) is used. The contours are consecutively resampled to the lower resolution grid. The sum of energy in the wavelet maps is normalized to the energy of the original image.

## **Distribution features definition**

The distribution features aim to quantify the spread of regional metastatic disease (metastatic lymph nodes LN) around the primary tumor (PT). This information is based purely on location and volumes of the nodes; no image intensity is taken into account. Here we present the summary of the distribution features and their definitions. The points within the primary tumor and lymph nodes are defined based on the clinical contours and a 1mm cubic grid. The clinical lymph node contour are additionally split into subcontours for single nodes (Figure 1). The distances between structures are defined as the distances between respective centers of the mass. The following features are then calculated.

1. Features based on the location of center of the mass

- Center of mass shift between primary tumor contour and contour encompassing primary tumor and lymph nodes, CM – center of mass.

1)

center of mass shift = $\sqrt{\left( {CM}_{PT}- {CM}_{PT+LN} \right)^{2}}$

- Distribution of distances between primary tumor and lymph nodes, *d_iPT_* – distance between the *ith* lymph node and primary tumor, *n* – number of nodes, *d_ij_* – distance between the *ith* and *jth* lymph node and primary tumor.

largest distance = $max(d_{iPT})$

mean distance = $\frac{1}{n}\sum_{i=1}^{n} d_{iPT}$

sum of distances = $\sum_{i=1}^{n} d_{iPT}$

1. sum of distances between primary tumor and lymph nodes distances normalized by the average distance between lymph nodes.

normalized sum of distances = $\frac{\sum_{i=n}^{n} d_{iPT}}{\frac{1}{n}\sum_{i=1}^{n} \sum_{j=i+1}^{n} d_{ij}}$

- Distribution of distances between primary tumor and lymph nodes weighted by lymph node’s volume, *v_i_* – volume of the *ith* lymph node.

largest weighted distance = $max(d_{iPT}v_{i})$

mean weighted distance = $\frac{1}{n}\sum_{i=1}^{n} d_{iPT}v_{i}$

- Distribution of distances between primary tumor and lymph nodes weighted by lymph node’s volume and normalized by tumor volume, *v_PT_* – volume of primary tumor.

normalized largest weighted distance = $max\left( \frac{d_{iPT}v_{i}}{v_{PT}} \right)$

normalized mean weighted distance = $\frac{1}{n}\sum_{i=1}^{n} \frac{d_{iPT}v_{i}}{v_{PT}}$

- Distribution of the smallest distances between the analyzed structures based on Kruskal algorithm [4] (see Figure 2), *mind_i_* – distribution of minimal distances, *µ_mind_* – mean of the distribution of minimal distances.

1. mean of the distribution of minimal distances

distance distribution mean = $\frac{1}{(n+1)}\sum_{k=1}^{n+1} {mind}_{k}$

1. variance of the distribution of minimal distances

distance distribution variance = $\frac{1}{(n+1)}\sum_{k=1}^{n+1} \left( {mind}_{k}- \mu_{mind} \right)^{2}$

1. Features based on all the points within contours

- Optimal number of clusters in kmeans clustering based on Calinski-Harabasz index and points within lymph nodes contour [5].


optimal cluster number

- Principal component analysis based on all the points within primary tumor and lymph nodes [1].


elongation = $\sqrt{\frac{minor principal axis}{major principal axis}}$


flatness = $\sqrt{\frac{least principal axis}{major principal axis}}$


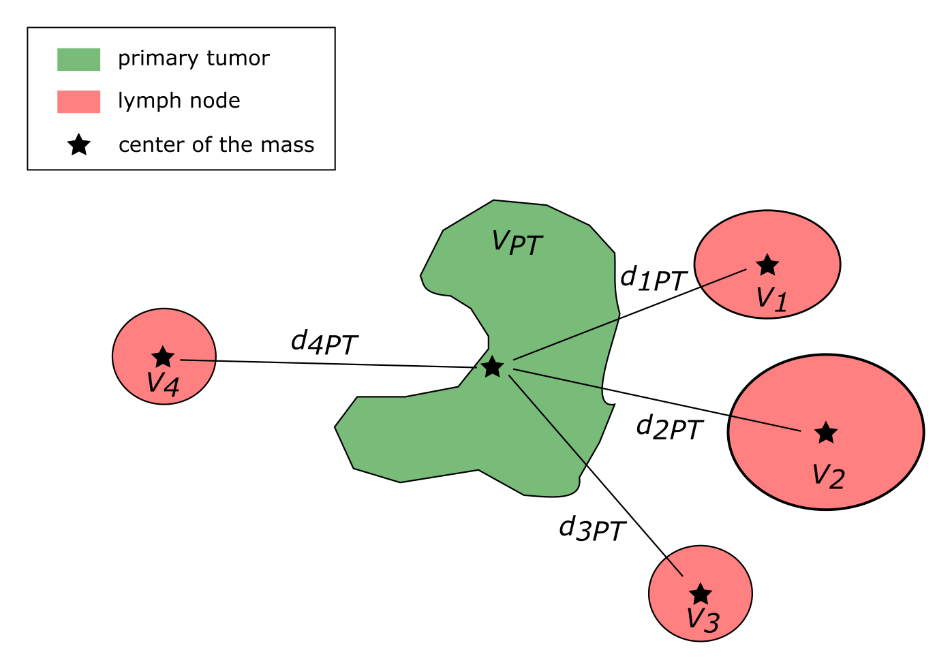


Figure 1. Scheme of the lymph nodes distribution around primary tumor; v*i* – volume of *ith* lymph node, *v_PT_* – volume of primary tumor, *d_iPT_* – distance between center of the mass of primary tumor and *ith* lymph node


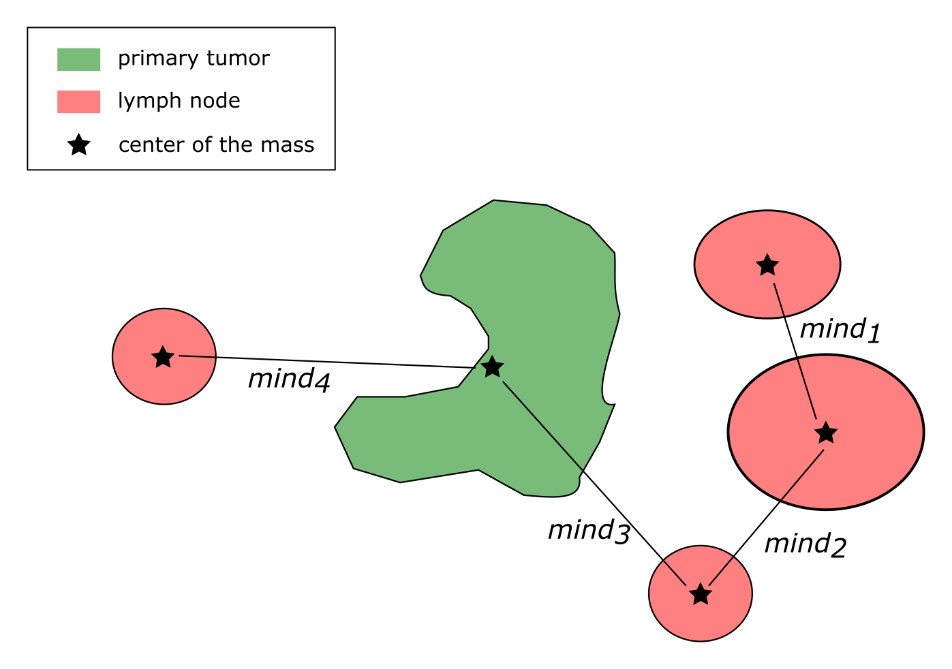


Figure 2. Scheme of the lymph nodes distribution around primary tumor additionally showing the shortest path between the structures

## **References**

1. Zwanenburg, A., et al. *Image biomarker standardisation initiative*. arXiv e-prints, 2016.

2. *Fractal Dimension Estimation Methods for Biomedical Images*, in *MATLAB - A Fundamental Tool for Scientific Computing and Engineering Applications - Volume 3*. 2012, InTech.

3. *Textural features for image classiification.* IEEE Trans Syst Man Cybern, 1973. **3**: p. 610-621.

4. Cormen, T.H., et al., *Introduction to algorithms*. 2009: MIT press.

5. Caliński, T. and J. Harabasz, *A dendrite method for cluster analysis.* Communications in Statistics-theory and Methods, 1974. **3**(1): p. 1-27.

# **Diagnostic performance of radiomic features – Left lung**

## Table 1 Diagnostic performance of radiomic features and visual assessment of HRCT-features. Gender, age and lung physiology- (GAP-) stage, number of patients (n), area under the curve (AUC) with bootstrapped 95% confidence intervals (CI).

| **Variable** | **GAP1 vs. GAP2 (augmented training set)** | | | **GAP1 vs. GAP2 (augmented testing set)** | | | **GAP1 vs. GAP2 (original dataset)** | |  |  |
| --- | --- | --- | --- | --- | --- | --- | --- | --- | --- | --- |
|  | **AUC** [95% CI] | **Sens.y (%)** | **Spec. (%)** | **AUC** [95% CI] | **Sens. (%)** | **Spec. (%)** | **AUC** [95% CI] | **Sens. (%)** | | **Spec. (%)** |
| M_homogenity_n.LHL | 1.00 [0.99 – 1.00] | 100 | 100 | 0.90 [0.79 – 1.00] | 100 | 75 | 0.96 [0.90 – 1.00] | 87 | | 100 |
| neighContrast.LHL | 1.00 [0.99 – 1.00] | 100 | 100 | 0.92 [0.82 – 1.00] | 100 | 75 | 0.90 [0.76 – 1.00] | 83 | | 93 |
| fractal_dim.LLL | 0.60 [0.27 – 0.54] | 40 | 100 | 0.55 [0.33 – 0.76] | 44 | 81 | 0.61 [0.34 – 0.88] | 50 | | 85 |
| M_correlation.HLL | 0.93 [0.88 – 0.99] | 100 | 82 | 0.75 [0.56 – 0.94] | 100 | 50 | 0.83 [0.63 – 1.00] | 67 | | 94 |
| correlation.HHL | 0.65 [0.51 – 0.79] | 92 | 55 | 0.60 [0.39 – 0.81] | 100 | 37 | 0.62 [0.45 – 0.79] | 100 | | 39 |
| sizeVar_n.LLH | 0.72 [0.61 – 0.84] | 58 | 82 | 0.72 [0.54 – 0.90] | 63 | 75 | 0.75 [0.50 – 0.99] | 67 | | 91 |
| M_homogenity_n.LHL + neighContrast.LHL | 1.00 [0.99 – 1.00] | 100 | 100 | 0.96 [0.89 – 1.00] | 94 | 94 | 0.96 [0.91 – 1.00] | 100 | | 91 |
| **neighContrast.LHL + M_correlation.HLL** | **1.00** [0.99 – 1.00] | **100** | **100** | **0.92** [0.82 – 1.00] | **100** | **75** | **0.90** [0.75 – 1.00] | **84** | | **93** |
